# Culture and National Well-Being: Should Societies Emphasize Freedom or Constraint?

**DOI:** 10.1371/journal.pone.0127173

**Published:** 2015-06-05

**Authors:** Jesse R. Harrington, Pawel Boski, Michele J. Gelfand

**Affiliations:** 1 University of Maryland, College Park, Maryland, United States of America; 2 University of Social Sciences and Humanities, Warsaw, Poland; University of Vienna, AUSTRIA

## Abstract

Throughout history and within numerous disciplines, there exists a perennial debate about how societies should best be organized. Should they emphasize individual freedom and autonomy or security and constraint? Contrary to proponents who tout the benefits of one over the other, we demonstrate across 32 nations that both freedom and constraint exhibit a curvilinear relationship with many indicators of societal well-being. Relative to moderate nations, very permissive and very constrained nations exhibit worse psychosocial outcomes (lower happiness, greater dysthymia, higher suicide rates), worse health outcomes (lower life expectancy, greater mortality rates from cardiovascular disease and diabetes) and poorer economic and political outcomes (lower gross domestic product per capita, greater risk for political instability). This supports the notion that a balance between freedom and constraint results in the best national outcomes. Accordingly, it is time to shift the debate away from either constraint or freedom and focus on both in moderation.

## Introduction

In fields as diverse as psychology, sociology, and political and economic philosophy, there is a long-standing debate concerning the best way to organize societies. Advocates of the importance of *freedom* claim that autonomy allows individuals to self-actualize and maximizes societal happiness and economic progress. Proponents of *constraint* insist that rules and regulations are critical for creating a secure and stable society that enables happiness and progress. Such debates are ancient in origin. They were found in Plato’s *Republic*, continued among Chinese and European philosophers, and extend into the modern era [1 – 5] (see also [6 – 9]). In the United States today, modern examples of this age-old tension include the controversy surrounding the Patriot Act in the years following 9/11 and the recent debates concerning the legitimacy of the National Security Agency’s mass surveillance techniques. However, despite much philosophizing, there has been surprisingly little empirical data brought to bear on this question. The age-old question remains: Does evidence favor freedom over constraint for societal well-being, or vice versa?

We argue that neither position is correct. Rather, *both* excessive freedom and excessive constraint are costly to societal well-being. In particular, overly constraining environments provide severe limitations for individual choice and necessitate constant behavioral self-monitoring, while overly permissive environments may promote lawlessness and normlessness (anomie), a lack of social predictability, and little to no rules that regulate extreme behavior and coordinate social action. This hypothesis, though untested, echoes theory in various disciplines, including political and economic philosophy [2, 3], sociology [1, 4], and psychology [5]. Durkheim [4], for instance, long theorized that high suicide rates would be produced in both very constraining and excessively individualistic and disorganized societies, though likely through different mechanisms; egoistic suicide results from perceptions of meaninglessness and a total lack of social integration produced by excessive individuality and a lack of purpose, while fatalistic suicide stems from a desire to die rather than live under constant state of oppressive control. Likewise, in psychology, Erich Fromm [5] made the claim that excessive freedom and the loosening of societal constraints leaves individuals without a sense order, producing high levels of anxiety and resulting in a pendulum shift to authoritarianism and conformity. More recently, Amitai Etzioni [1] theorized that society is enriched when both autonomy and order are blended together and that an emphasis on either alone is problematic for societal functioning.

Despite the notion that a balance of permissiveness and constraint may produce optimal societal outcomes, there has been no empirical test of this proposition to date. In this study, we test the linear and curvilinear effects of societal permissiveness versus constraint on a wide range of societal indicators, including psychosocial (happiness, incidence of dysthymia—or low-level, chronic depression [10]—and suicide rates), health (mortality rate from cardiovascular disease and diabetes and life expectancy), and economic/political (gross domestic product per capita and risk for political instability) outcomes.

## Materials and Methods

Societal permissiveness versus constraint was indexed through well-validated indices of cultural looseness versus tightness [11]. Loose societies have weak social norms, are permissive, and have a high tolerance for deviant behavior. Tight societies have strong social norms, are restrictive, and have a low tolerance for deviance. In all, tight societies limit freedom and engender constraint while loose societies afford greater permissiveness. Nations vary on this dimension across a continuum from very loose to very tight. Previous research by Gelfand and colleagues [11] provided the tightness-looseness scores used in the present research. Thirty-two nations from their data were represented in this study, including Australia, Austria, Belgium, Brazil, Estonia, France, Germany (tightness scores were averaged for the former East and West), Greece, Hong Kong, Hungary, Iceland, India, Israel, Italy, Japan, Malaysia, Mexico, Netherlands, New Zealand, Norway, Pakistan, China, Poland, Portugal, Singapore, South Korea, Spain, Turkey, Ukraine, United Kingdom, USA, and Venezuela. Individuals in all nations were asked to report their agreement or disagreement to questions such as whether there are many social norms that people are supposed to abide by in their country, whether there are clear expectations for how people should act in most situations, whether people have a great deal of freedom in deciding how they can act (reverse scored), whether one will be disapproved of if one acts in an inappropriate way, and whether people almost always comply with social norms. There were high levels of inter-rater agreements in all countries and the scale has convergent and divergent validity (see [11]).

Psychosocial outcomes are associated with issues of psychological and social health and well-being. Theoretically, if very permissive societies and very restrictive societies produce a high degree of stressors—as theorized by Durkheim, Fromm, and Etzioni—they should produce lower happiness, greater dysthymia, and a higher suicide rate. In contrast, those societies that are more moderate on constraint-permissiveness should exhibit greater happiness, less dysthymia, and lower suicide. *Happiness* was assessed using the happiness index compiled by ASEP/JDS [12], with scores taken from the World Values Survey, the European Values Survey, Latinobarómetro, and the International Social Survey Program. *Prevalence of dysthymia* was assessed with data acquired from the World Health Organization’s 2010 Global Burden of Disease Study [13]. Finally, *suicide rate* was assessed with data taken from a World Health Organization report [14].

Health outcomes deal with the physical well-being of a nation’s citizens. The higher degree of stressors theorized to be present in very permissive and very restrictive societies should produce poorer health outcomes, including a greater mortality rate associated with cardiovascular disease and diabetes and lower life expectancy. Notably, research has shown that environmental stressors induce more severe outcomes and symptoms in diabetics [15]. *Mortality rate from cardiovascular diseases and diabetes* was assessed with data from the World Health Organization [16]. *Life expectancy in years* was assessed with data from the CIA World Factbook [17].

Economic outcomes refer to the degree of material and monetary wealth of a nation. Theoretically, very loose societies lack the ability to coordinate social action whereas very tight societies have high repression, both of which should negatively relate to efficiency and production. Consequently, we suspect that GDP per capita will be lower in both very constrained and very permissive nations. We acquired data on *gross domestic product per capita* from the CIA World Factbook [18]. We also anticipated that very tight and very loose nations would have a higher risk for political instability, or episodes involving disruption of normal public and private life that are commonly accompanied by some degree of violence. Nations that are highly suppressed or, in contrast, highly disorganized should result in greater social stresses, as well as lower life satisfaction and quality of life. This may result in a higher degree of political action and, consequently, risk for greater political instability and social unrest. We acquired *risk for political instability* scores from The Economist’s Intelligence Unit for the years 2009–2010 [19], which accounts for multiple social and economic risk factors.

We anticipated that the relationship between permissiveness (looseness) and constraint (tightness) and the above outcomes would exhibit a curvilinear relationship, such that very tight and very loose nations have worse outcomes relative to nations intermediate on tightness-looseness. We used stepwise multiple regression to evaluate these hypotheses. Step 1 examined the linear effect of tightness-looseness on the outcome variable in question. Step 2 introduced a quadratic term to account for the hypothesized curvilinear effect. Below we report linear and curvilinear effects for each of the psychosocial, health, economic, and political outcomes. We also report the results of an analysis using a generalized index of these outcomes that was derived from factor analysis. As wealth disparity and other prominent cultural dimensions may also influence these variables, we also control for GINI [20] and individualism [21] in secondary analyses. Note that differing degrees of freedom across different analyses reflect the fact that some countries lacked data for some analyses.

## Results

### National psychosocial outcomes

**Fig 1:**
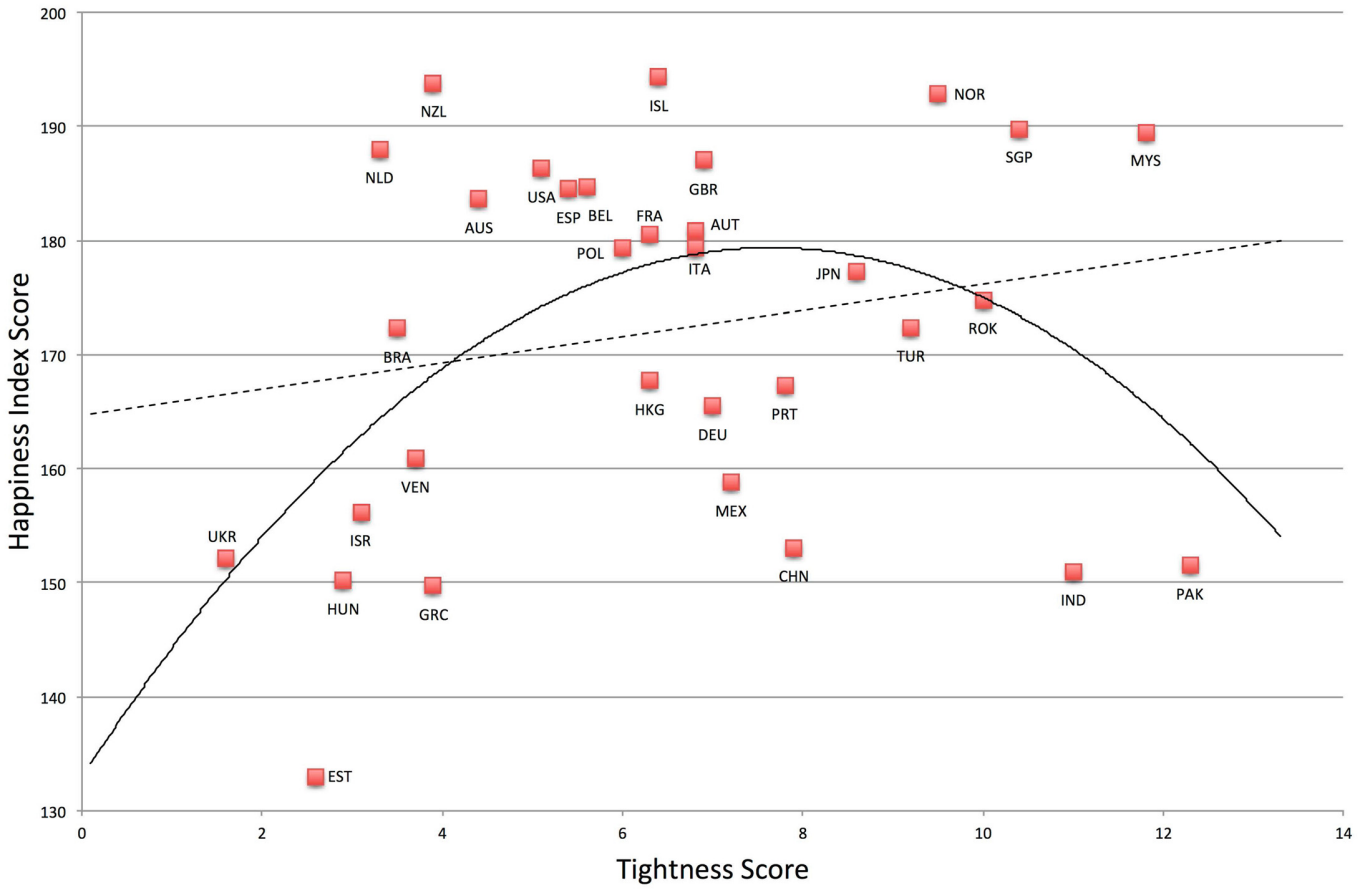
Relationship between Tightness-Looseness and Happiness.

**Fig 2:**
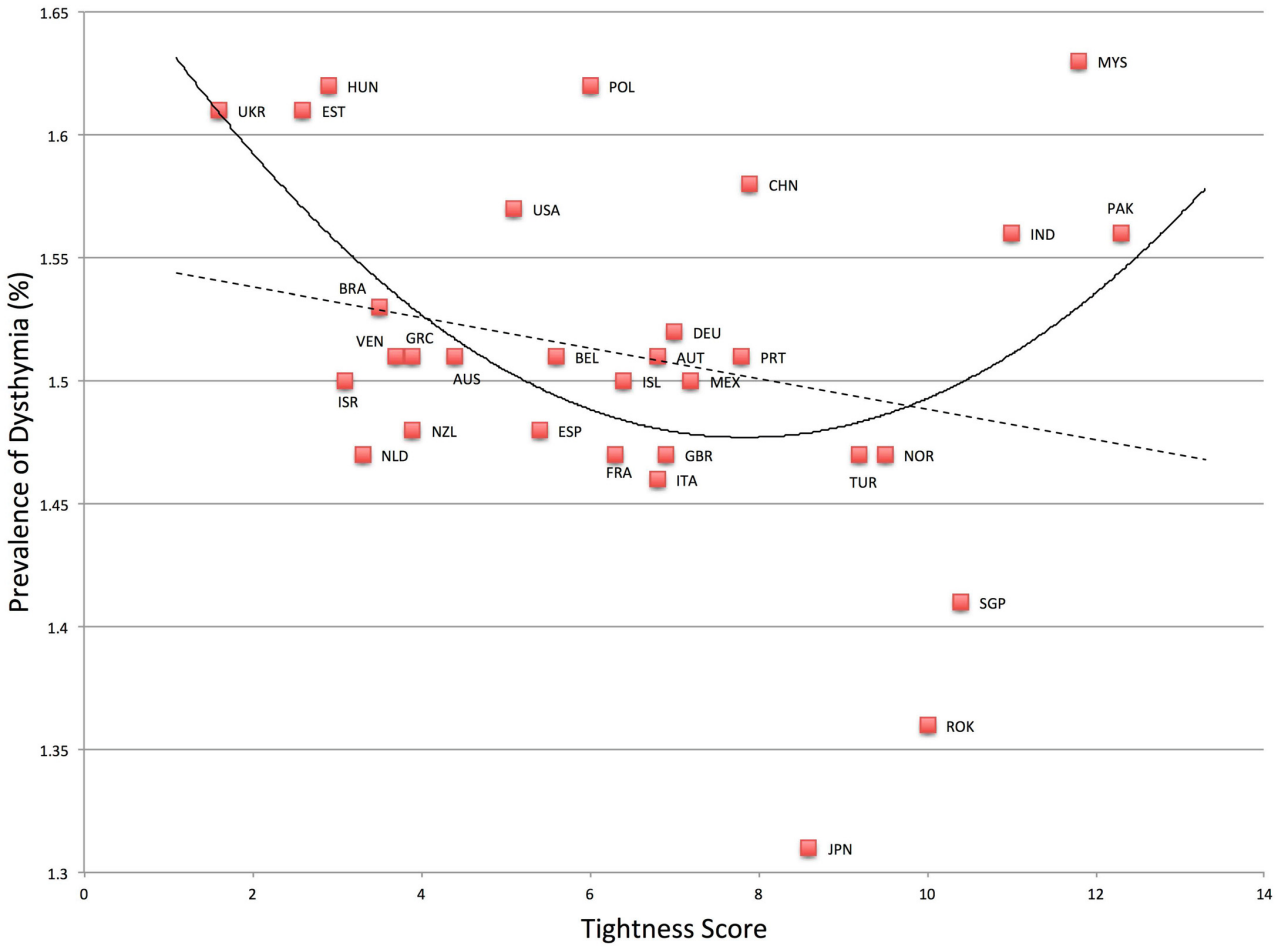
Relationship between Tightness-Looseness and Dysthymia.

**Fig 3:**
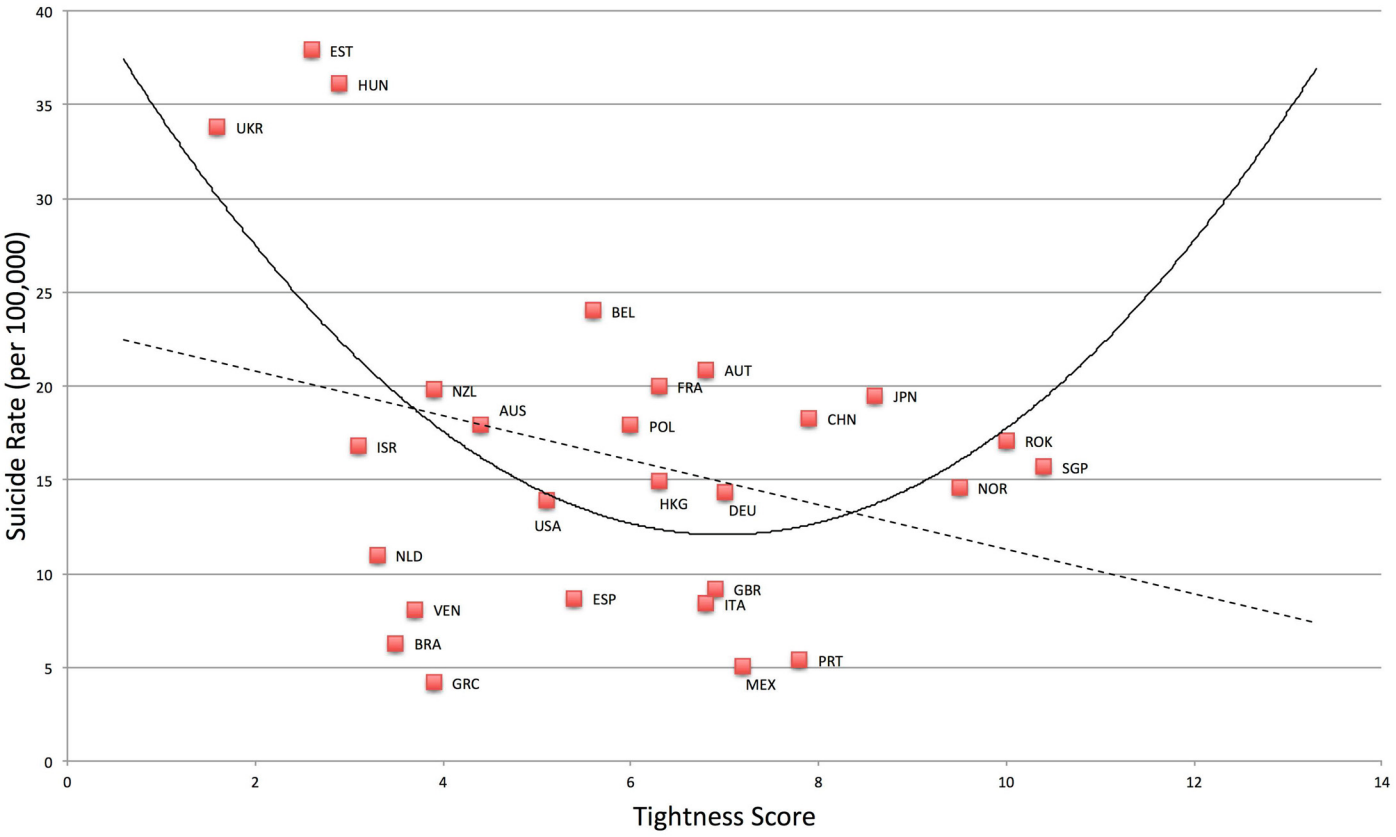
Relationship between Tightness-Looseness and Suicide.

As predicted, nations that are very tight and very loose have low happiness, *F*(2, 29) = 3.91, *p* = .03, *R* ^*2*^ = .21. See Fig 1. Compared to the linear model, *F*(1, 30) = 1.25, *p* = .27, *R* ^*2*^ = .04, the quadratic model was a significant improvement, *F*-change (1, 29) = 6.34, *p* = .02, *R* ^*2*^ change = .17. Dysthymia was also found to be higher in both very tight and very loose nations, *F*(2, 28) = 3.86, *p* = .03, *R* ^*2*^ = .22. See Fig 2. Relative to the linear model, *F*(1, 29) = 1.87, *p* = .18, *R* ^*2*^ = .06, the quadratic model was a significant improvement, *F*-change (1, 28) = 5.55, *p* = .03, *R* ^*2*^ change = .16. Suicide rate was also higher in very tight and very loose nations, *F*(2, 24) = 4.00, *p* = .03, *R* ^*2*^ = .25. See Fig 3. Compared to the linear model, *F*(1, 25) = 2.76, *p* = .11, *R* ^*2*^ = .06, the quadratic model was a significant improvement, *F*-change (1, 24) = 4.81, *p* = .04, *R* ^*2*^ change = .15.

### National health outcomes

**Fig 4:**
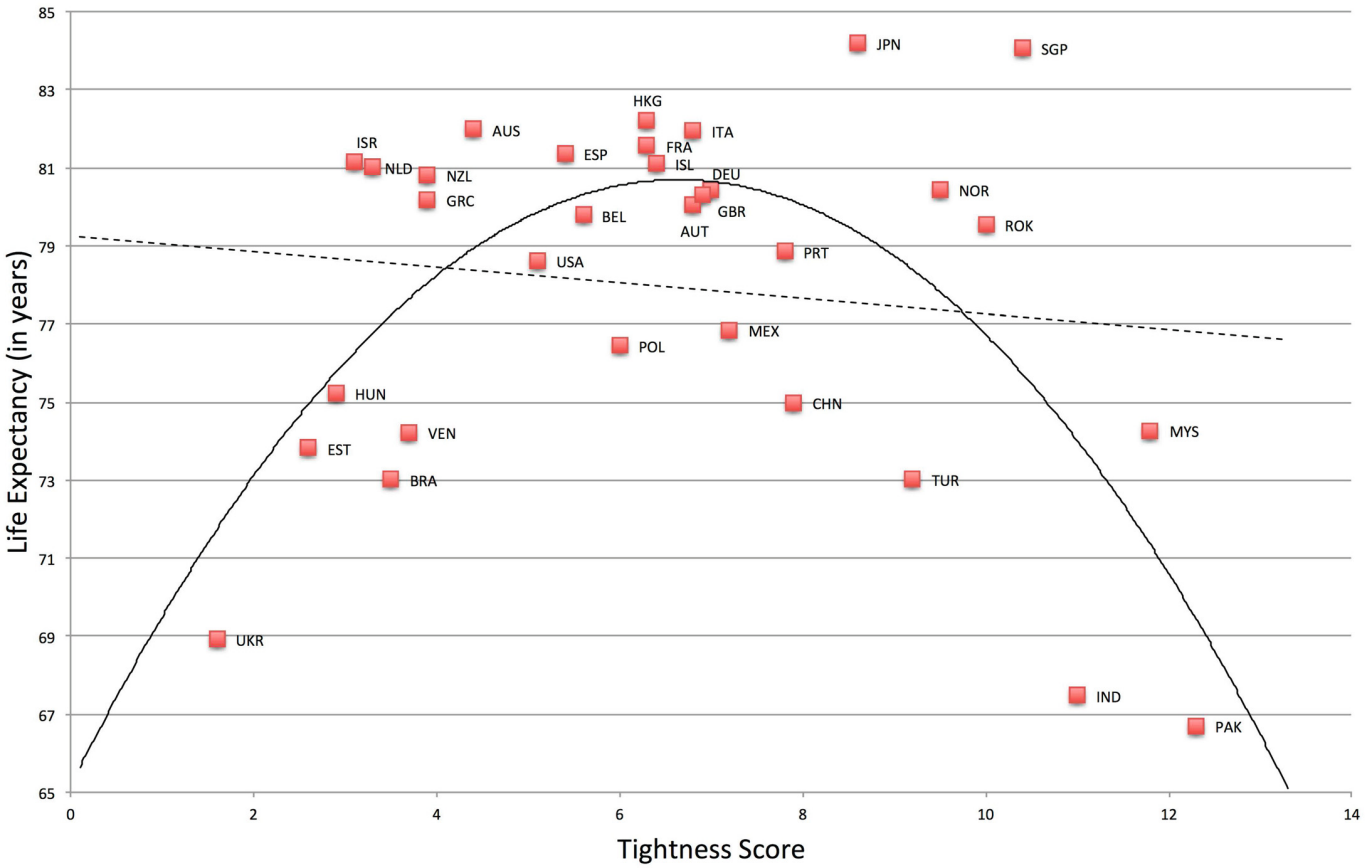
Relationship between Tightness-Looseness and Life Expectancy.

**Fig 5:**
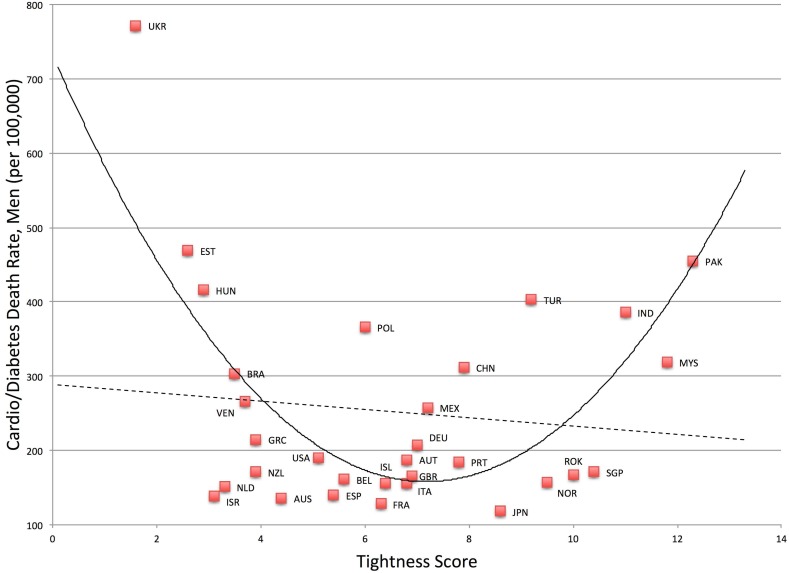
Relationship between Tightness-Looseness and Cardio/Diabetes Death Rates in Men.

**Fig 6:**
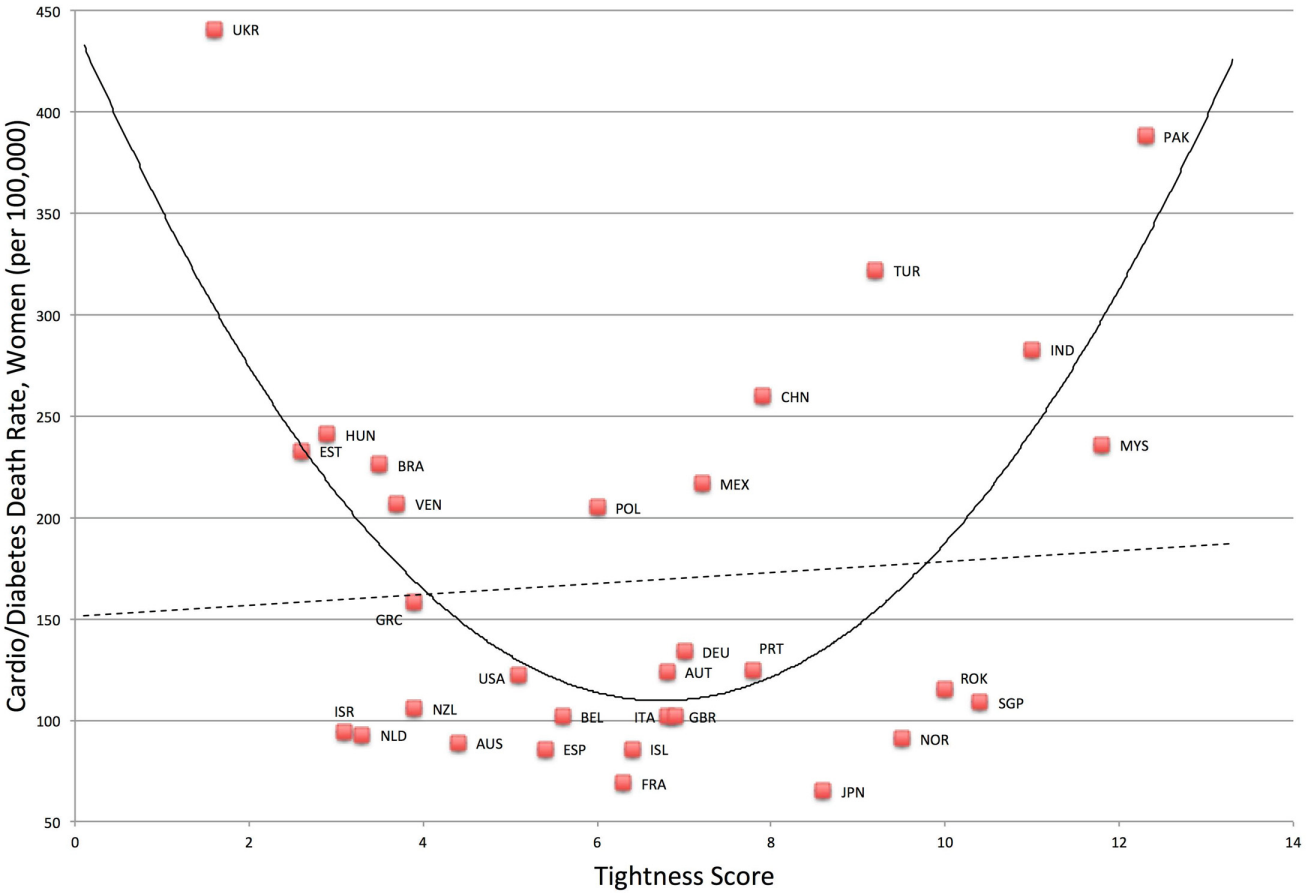
Relationship between Tightness-Looseness and Cardio/Diabetes Death Rates in Women.

Results show that both very tight and very loose nations have low life expectancy *F*(2, 29) = 11.41, *p* < .001, *R* ^*2*^ = .44. See Fig 4. Compared to the linear model, *F*(1, 30) = .46, *p* = .50, *R* ^*2*^ = .02, the quadratic model was a significant improvement, *F*-change (1, 29) = 22.04, *p* < .001, *R* ^*2*^ change = .42. Likewise, mortality rates for cardiovascular disease and diabetes were also greater in very tight and very loose nations for both men, *F*(2, 28) = 11.23, *p* < .001, *R* ^*2*^ = .45, and women, *F*(2, 28) = 10.09, *p* = .001, *R* ^*2*^ = .42. See Figs 5 and 6. Compared to the linear models, (men) *F*(1, 29) = .37, *p* = .55, *R* ^*2*^ = .01, (women) *F*(1, 29) = .19, *p* = .67, *R* ^*2*^ = .01, the quadratic models were a significant improvement, (men) *F*-change (1, 28) = 21.81, *p* < .001, *R* ^*2*^ change = .43, (women) *F*-change (1, 28) = 19.87, *p* < .001, *R* ^*2*^ change = .41.

### National economic and political outcomes

**Fig 7:**
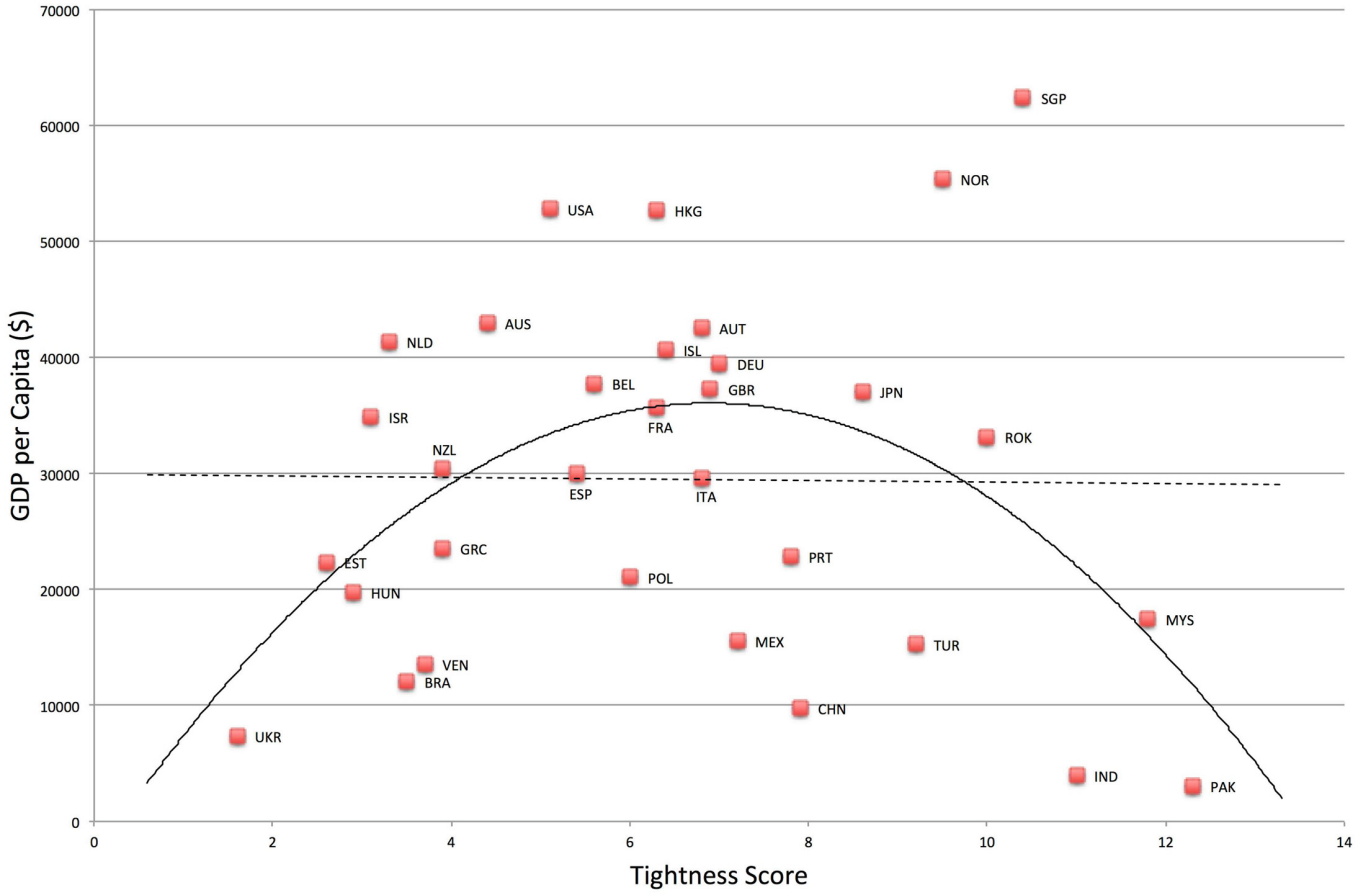
Relationship between Tightness-Looseness and GDP per Capita.

**Fig 8:**
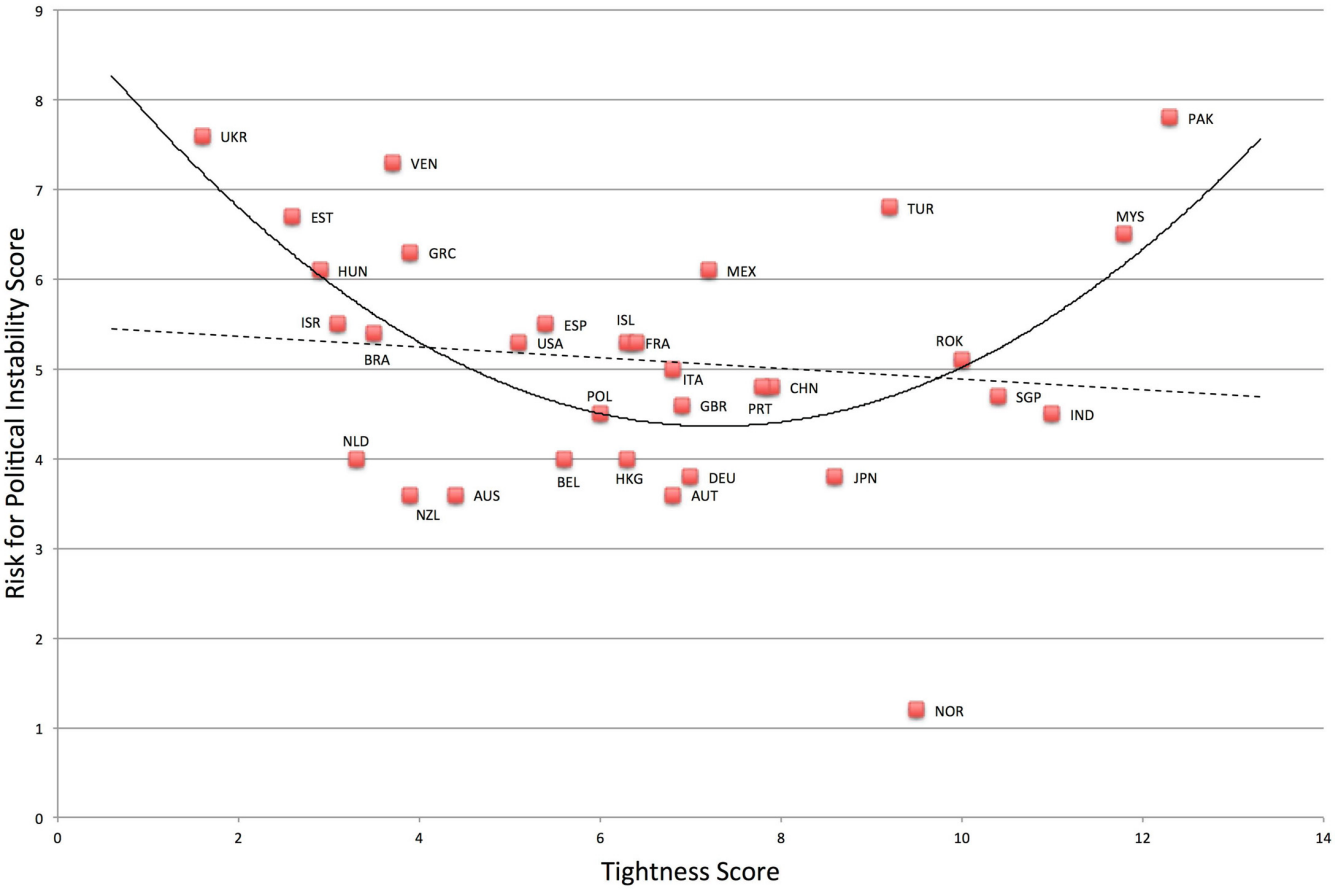
Relationship between Tightness-Looseness and Political Instability.

Gross domestic product (GDP) per capita was lower in both very tight and very loose nations, *F*(2, 29) = 3.79, *p* = .03, *R* ^*2*^ = .21. See Fig 7. Relative to the linear model, *F*(1, 30) = .004, *p* = .95, *R* ^*2*^ = .0001, the quadratic model was a significant improvement, *F*-change (1, 29) = 7.58, *p* = .01, *R* ^*2*^ change = .21. In addition, risk for political instability is much higher in both very tight and very loose nations, *F*(2, 29) = 6.33, *p* = .005, *R* ^*2*^ = .30. See Fig 8. Relative to the linear model, *F*(1, 30) = .45, *p* = .51, *R* ^*2*^ = .02, the quadratic model was a significant improvement, *F*-change (1, 29) = 12.05, *p* = .002, *R* ^*2*^ change = .29.

### Composite Score

**Fig 9:**
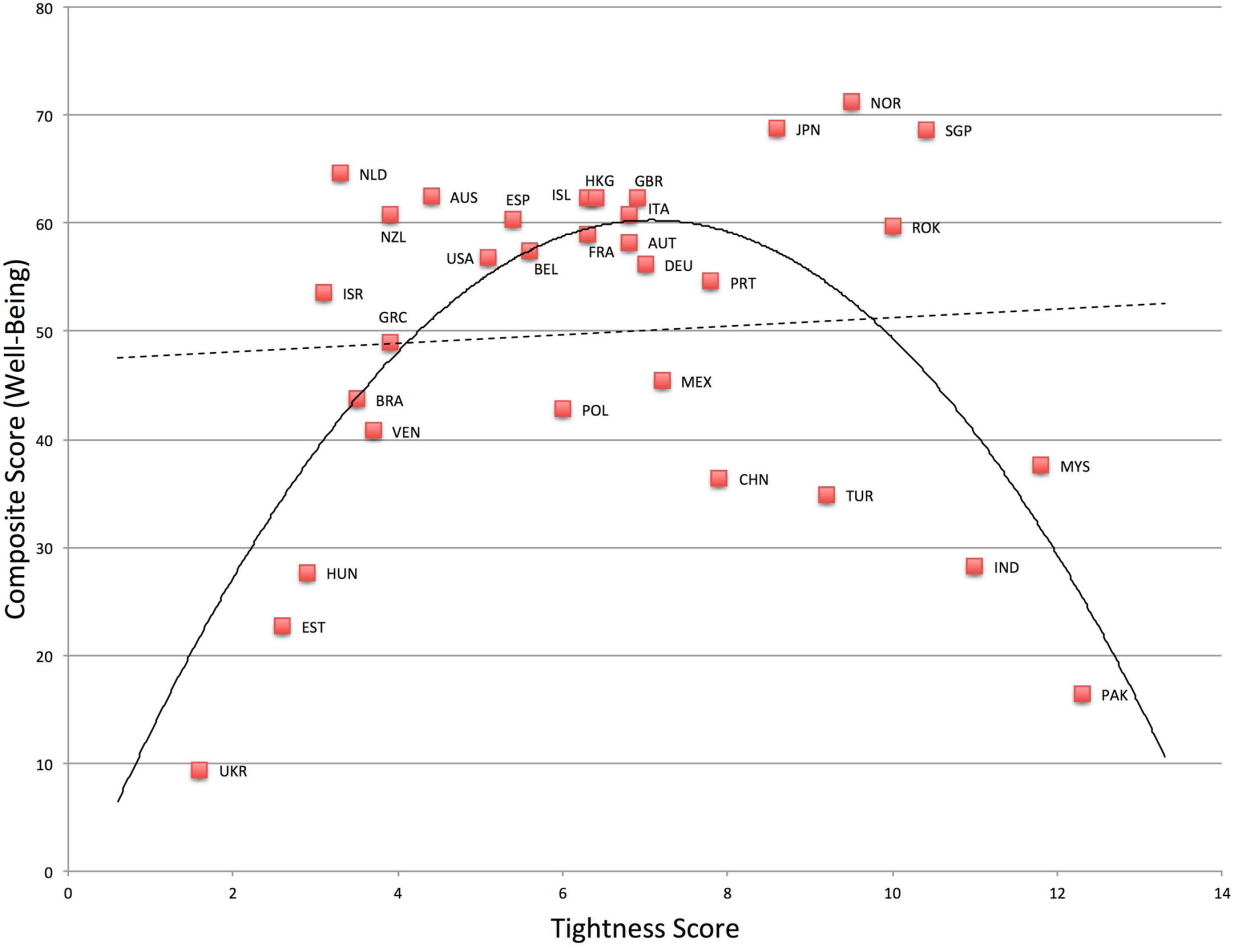
Relationship between Tightness-Looseness and Composite Score.

For an overarching test of the theory, we created a composite score of all 8 variables noted above. All 8 variables were moderately correlated, internally consistent (α = .91), and loaded highly on a single factor (see the S1 File and S1 and S2 Tables for full details). Some variables were reversed so that higher composite scores were indicative of greater well-being. Nations that were very tight or very loose exhibited lower composite scores, *F*(2, 29) = 12.72, *p* < .001, *R* ^*2*^ = .47. See Fig 9. Compared to the linear model, *F*(1, 30) = .15, *p* = .70, *R* ^*2*^ = .01, the quadratic model was a significant improvement, *F*-change (1, 29) = 25.17, *p* < .001, *R* ^*2*^ change = .46.

### Controls and Model Comparison

The results of these analyses do not change substantially when controlling for income disparity (GINI) and cultural individualism (see S4, S5, S6, S7, S8, S9, S10 and S11 Tables; one exception is happiness, which was only marginally significant with the controls added, see S3 Table). In addition, Corrected Akaike Information Criteria (AICc) demonstrate that the quadratic models are a better fit for the data relative to the linear models (see S12 Table).

## Discussion

To date, this research is the most extensive examination of the relationship between freedom and constraint and a wide array of societal outcomes. Our results clearly support the notion that excessive constraint (tightness) and excessive freedom (looseness) contribute to poorer psychosocial, health, and economic/political outcomes, as well as lower overall well-being at the national level. Relative to more moderate nations, both very permissive and very constrained nations exhibit lower happiness, greater dysthymia, higher suicide rates, lower life expectancy, greater mortality rates from cardiovascular disease and diabetes, lower gross domestic product per capita, and a higher risk for political instability.

These disadvantages associated with both extremes of freedom and constraint may stem from a common mechanism, namely the inability to control oneself and one’s environment, which has been identified as a core human need (e.g., [22, 23]). Excessive constraint severely limits individual choice and requires constant self-monitoring [11], while excessive freedom provides fewer guiding rules, greater social disorganization, and greater social unpredictability. Both can undermine perceptions of control, either by restricting autonomy in the case of excessive constraint or, in the case of excessive freedom, by not being able to coordinate because of the anomie and randomness in one’s environment. On the other hand, more balanced, intermediate approaches may promote a symbiotic relationship between constraint and freedom that fosters a sense of control and produces optimal psychosocial, health, and economic outcomes [1]. Put differently, a moderate degree tightness-looseness allows for both choice and a normative structure that permits prediction and coordinated action. It remains up to future research to determine if this mechanism is in fact contributing to the curvilinear relationships documented in this research.

Being able to diagnose extreme levels of tightness-looseness is not only helpful for understanding societal well-being; it may also help to predict radical pendulum shifts that occur these extremes. For example, when autocratic governments were overthrown after the Arab Spring, we witnessed a shift from very tight societal control to normlessness, anomie, and disorganization, which ironically created the need for strong leaders—a phenomenon that might be coined “autocratic recidivism.” Such pendulum shifts have also been observed elsewhere. For example, after the dissolution of the USSR in 1991, 51% of Russians supported democracy while only 39% supported strong leadership [24]; this has shifted dramatically over time, with 57% of Russians supporting strong leadership and only 32% supporting democracy in 2012 [25]. In addition, the percentage of Russians who prefer the freedom to pursue life goals unimpeded by state interference has dropped from 53% to 26% between 1991 and 2011 [26]. This illustrates clear pendulum shifts in this context. The Ukraine is another example of a nation that shifted toward greater anomie following the dissolution of the USSR, resulting in more chaos, corruption, and lawlessness—all risk factors for a pendulum shift toward tightness. In all, this has important implications for geopolitical stability, as well as the behaviors, outcomes, and social and psychological motivations for individuals within these societies.

## Limitations and Future Directions

As with all research, there are limitations to the present study. First, only 32 nations were included in the present analysis due to the availability of tightness-looseness scores from Gelfand and colleagues [11]. Future research incorporating more national level data on both tightness-looseness and the variables included in this study would help to extend this work to more countries around the globe. Moreover, the national tightness-looseness scores used in this research are based upon a self-report descriptive norm approach to assessing culture. In other words, they asked participants to assess how most people within their nation would perceive the social norm strength and deviance tolerance of their respective society. While numerous studies have demonstrated the validity of this approach [27 – 32] and the researchers found high agreement, or convergent perceptions, between individuals in each nation [11], others have criticized this general approach as tapping into inaccurate national stereotypes [33, 34] (but see [35, 36, 37, 38]). Future research would benefit from employing additional methods of assessing permissiveness and constraint. We also note that our work focused on the strength of social norms in a society, which is independent from the strength of institutions. For example, tightness-looseness is unrelated to the rule of law (*r*(30) = -.09, *p* = .64; see the World Justice Project’s Rule of Law Index [39]), thus it would be interesting to examine how they interact to affect societal functioning in future research. Finally, some of the criterion variables included in the present research are self-reports (e.g., happiness). As some have suggested [40], comparing self-ratings across nations can be problematic. However, many of the variables in this study are based on more objective criteria (e.g., suicide rates, GDP per capita, life expectancy, morality rates due to cardiovascular diseases and diabetes), lending credence to our theory.

## Conclusion

Across the span of human history, there has been a longstanding debate concerning the relative benefits of constraint versus freedom. Our results suggest that moderate levels of societal tightness-looseness, or a balance of freedom and constraint, are associated with optimal psychosocial, health, and economic/political outcomes. Accordingly, it is time to shift the debate away from constraint versus freedom and focus on both in moderation.

## Supporting Information

S1 File Supplemental Information. (DOCX)

S1 Data File Data for all Variables and Nations. (XLSX)

S1 Table Correlations between Well-Being Index Variables. (DOCX)

S2 Table Factor Loadings for Well-Being Index Variables. (DOCX)

S3 Table Happiness: Regression Results Controlling for GINI and Individualism. (DOCX)

S4 Table Dysthymia: Regression Results Controlling for GINI and Individualism. (DOCX)

S5 Table Suicide Rate: Regression Results Controlling for GINI and Individualism. (DOCX)

S6 Table Life Expectancy: Regression Results Controlling for GINI and Individualism. (DOCX)

S7 Table Male Mortality Rate for Cardiovascular Diseases and Diabetes: Regression Results Controlling for GINI and Individualism. (DOCX)

S8 Table Female Mortality Rate for Cardiovascular Diseases and Diabetes: Regression Results Controlling for GINI and Individualism. (DOCX)

S9 Table GDP per Capita: Regression Results Controlling for GINI and Individualism. (DOCX)

S10 Table Political Instability Index: Regression Results Controlling for GINI and Individualism. (DOCX)

S11 Table Well-Being Composite Score: Regression Results Controlling for GINI and Individualism. (DOCX)

S12 Table Corrected Akaike Information Criteria Comparisons. (DOCX)
