# Supplementary material for: Culture and National Well-Being: Should Societies Emphasize Freedom or Constraint?
Source: PLoS One. 2015 Jun 5;10(6):e0127173. doi: 10.1371/journal.pone.0127173 (PMC4457878; doi:10.1371/journal.pone.0127173)
Supplement: S1 File — (DOCX) [file pone.0127173.s001.docx]

**Supplemental**

We conducted a factor analysis and combined all 8 of the variables examined previously to produce a composite score of well-being. All variables were moderately correlated (see Table S1) and internally consistent (α = .91). The Kaiser-Meyer-Olkin Measure of Sampling was equal to 0.81—exceeding the recommended value of 0.60 [1, 2]—and Bartlett’s Test of Sphericity was statistically significant, χ^2^(28) = 179.68, *p* < .001, indicating that the data were suitable for factor analysis [3]. Exploratory Factor Analysis using Maximum Likelihood Estimation produced a single factor solution that accounted for 60.70% of the variance, as well as a scree plot that plateaued after the first factor. All variables loaded highly on this single factor (see Table S2) with the lowest being suicide rate at .41. As expected, evidence suggests that all 8 variables are representative of a single underlying construct; namely, well-being.

In order to produce a composite score of well-being using these 8 items, we first reversed variables indicative of lower well-being, including incidence of dysthymia, suicide rate, mortality rate due to cardiovascular disease and diabetes for both men and women, and political instability. Next, we standardized and summed all scores for each nation in the study. We further transformed these scores in the following manner: we divided each score by the total number of variables (i.e., 8), then multiplied each score by 20 and added the result to 50. This produced positive and easily interpretable scores for Figure 9. However, some nations (Hong Kong, Iceland, India, Malaysia, Pakistan, and Turkey) had missing data for one or two variables. Consequently, we only divided their scores by the amount of variables used to produce the initial standardized composite score (e.g., 7 in the case of Iceland).

**References**

1. Kaiser H (1970) A second generation Little Jiffy. Psychometrika, 35:401–415.

2. Kaiser H (1974) An index of factorial simplicity. Psychometrika, 39:31–36.

3. Bartlett MS (1954) A note on the multiplying factors for various chi square

approximations. Journal of the Royal Statistical Society, 16(Series B):296–298.
