# Supplementary material for: Culture and National Well-Being: Should Societies Emphasize Freedom or Constraint?
Source: PLoS One. 2015 Jun 5;10(6):e0127173. doi: 10.1371/journal.pone.0127173 (PMC4457878; doi:10.1371/journal.pone.0127173)
Supplement: S1 Table — (DOCX) [file pone.0127173.s003.docx]

**Table S1.** Correlations between Well-Being Index Variables

| Variables | 1 | 2 | 3 | 4 | 5 | 6 | 7 | 8 |
| --- | --- | --- | --- | --- | --- | --- | --- | --- |
| 1. Happiness | - |  |  |  |  |  |  |  |
| 2. Incidence of Dysthymia | -.42^*^ | - |  |  |  |  |  |  |
| 3. Suicide Rate | -.33^†^ | .38^*^ | - |  |  |  |  |  |
| 4. Life Expectancy | .59^**^ | -.63^**^ | -.34^†^ | - |  |  |  |  |
| 5. Cardiovascular Disease/Diabetes  Morality Rate, Men | -.62^**^ | .65^**^ | .57^**^ | -.85^**^ | - |  |  |  |
| 6. Cardiovascular Disease/Diabetes  Morality Rate, Women | -.63^**^ | .60^**^ | .40^**^ | -.93^**^ | .94^**^ | - |  |  |
| 7. GDP per Capita | .63^**^ | -.49^**^ | -.08 | .83^**^ | -.69^**^ | -.80^**^ | - |  |
| 8. Political Instability | -.56^**^ | .39^**^ | .14 | -.61^**^ | .62^**^ | .67^**^ | -.66^**^ | - |

* *p* < .01; ** *p* < .01; ^†^*p* < .10
