# Supplementary material for: Culture and National Well-Being: Should Societies Emphasize Freedom or Constraint?
Source: PLoS One. 2015 Jun 5;10(6):e0127173. doi: 10.1371/journal.pone.0127173 (PMC4457878; doi:10.1371/journal.pone.0127173)
Supplement: S2 Table — (DOCX) [file pone.0127173.s004.docx]

**Table S2.** Factor Loadings for Well-Being Index Variables

| Variables | Factor Loadings |
| --- | --- |
| Happiness | -.64 |
| Incidence of Dysthymia | .61 |
| Suicide Rate | .41 |
| Life Expectancy | -.94 |
| Cardiovascular Disease/Diabetes Morality Rate, Men | .94 |
| Cardiovascular Disease/Diabetes Morality Rate, Women | .99 |
| GDP per Capita | -.81 |
| Political Instability | .67 |
