# Supplementary material for: Culture and National Well-Being: Should Societies Emphasize Freedom or Constraint?
Source: PLoS One. 2015 Jun 5;10(6):e0127173. doi: 10.1371/journal.pone.0127173 (PMC4457878; doi:10.1371/journal.pone.0127173)
Supplement: S3 Table — (DOCX) [file pone.0127173.s005.docx]

**Table S3.** Happiness: Regression Results Controlling for GINI and Individualism

| Happiness | Model 1 | | | Model 2 | | | Model 3 | | | Model 4 | | |
| --- | --- | --- | --- | --- | --- | --- | --- | --- | --- | --- | --- | --- |
|  | *B* | *SE B* | *β* | *B* | *SE B* | *β* | *B* | *SE B* | *Β* | *B* | *SE B* | *β* |
| GINI | -.10 | .38 | -.05 | .40 | .41 | .20 | .51 | .37 | .25 | .38 | .36 | .19 |
| Individualism |  |  |  | .31 | .13 | .47 | .48 | .13 | .73** | .41 | .13 | .63** |
| Tightness |  |  |  |  |  |  | 2.87 | 1.01 | .50** | 10.28 | 4.06 | 1.79* |
| Tightness^2^ |  |  |  |  |  |  |  |  |  | -.55 | .29 | -1.36§ |
| df1, df2 | 1, 29 | | | 2, 28 | | | 3, 27 | | | 4, 26 | | |
| *F* | .06 | | | 2.80 | | | 5.08 | | | 5.04 | | |
| *R^2^* | .01 | | | .17 | | | .36 | | | .44 | | |
| *R^2^* Change |  | | | .16 | | | .19 | | | .08 | | |
| *F* for *R^2^* Change |  | | | 5.54* | | | 8.19** | | | 3.52§ | | |

* *p* < .05. ** *p* < .01. § *p* < .10.
