# Supplementary material for: Culture and National Well-Being: Should Societies Emphasize Freedom or Constraint?
Source: PLoS One. 2015 Jun 5;10(6):e0127173. doi: 10.1371/journal.pone.0127173 (PMC4457878; doi:10.1371/journal.pone.0127173)
Supplement: S4 Table — (DOCX) [file pone.0127173.s006.docx]

**Table S4.** Dysthymia: Regression Results Controlling for GINI and Individualism

| Dysthymia | Model 1 | | | Model 2 | | | Model 3 | | | Model 4 | | |
| --- | --- | --- | --- | --- | --- | --- | --- | --- | --- | --- | --- | --- |
|  | *B* | *SE B* | *β* | *B* | *SE B* | *β* | *B* | *SE B* | *β* | *B* | *SE B* | *Β* |
| GINI | .01 | .01 | .02 | .01 | .01 | .08 | .01 | .01 | .05 | .01 | .01 | .15 |
| Individualism |  |  |  | .01 | .01 | .12 | .01 | .01 | -.02 | .01 | .01 | .13 |
| Tightness |  |  |  |  |  |  | -.01 | .01 | -.27 | -.06 | .02 | -2.18* |
| Tightness^2^ |  |  |  |  |  |  |  |  |  | .01 | .01 | 2.02* |
| df1, df2 | 1, 28 | | | 2, 27 | | | 3, 26 | | | 4, 25 | | |
| *F* | .01 | | | .14 | | | .60 | | | 1.90 | | |
| *R^2^* | .01 | | | .01 | | | .07 | | | .23 | | |
| *R^2^* Change |  | | | - | | | .06 | | | .16 | | |
| *F* for *R^2^* Change |  | | | - | | | 1.52 | | | 5.49* | | |

* *p* < .05. ** *p* < .01. § *p* < .10.
