# Supplementary material for: Culture and National Well-Being: Should Societies Emphasize Freedom or Constraint?
Source: PLoS One. 2015 Jun 5;10(6):e0127173. doi: 10.1371/journal.pone.0127173 (PMC4457878; doi:10.1371/journal.pone.0127173)
Supplement: S5 Table — (DOCX) [file pone.0127173.s007.docx]

**Table S5.** Suicide Rate: Regression Results Controlling for GINI and Individualism

| Suicide Rate | Model 1 | | | Model 2 | | | Model 3 | | | Model 4 | | |
| --- | --- | --- | --- | --- | --- | --- | --- | --- | --- | --- | --- | --- |
|  | *B* | *SE B* | *β* | *B* | *SE B* | *β* | *B* | *SE B* | *β* | *B* | *SE B* | *β* |
| GINI | -.46 | .21 | -.42* | -.40 | .24 | -.36 | -.43 | .24 | -.38§ | -.33 | .23 | -.30 |
| Individualism |  |  |  | .04 | .08 | .12 | -.01 | .09 | -.02 | .05 | .08 | .13 |
| Tightness |  |  |  |  |  |  | -.82 | .67 | -.26 | -6.35 | 2.60 | -2.02* |
| Tightness^2^ |  |  |  |  |  |  |  |  |  | .41 | .19 | 1.85* |
| df1, df2 | 1, 24 | | | 2, 23 | | | 3, 22 | | | 4, 21 | | |
| *F* | 5.10 | | | 2.63 | | | 2.29 | | | 3.22 | | |
| *R^2^* | .18 | | | .19 | | | .24 | | | .38 | | |
| *R^2^* Change |  | | | .01 | | | .05 | | | .14 | | |
| *F* for *R^2^* Change |  | | | .30 | | | 1.51 | | | 4.81* | | |

* *p* < .05. ** *p* < .01. § *p* < .10.
