# Supplementary material for: Culture and National Well-Being: Should Societies Emphasize Freedom or Constraint?
Source: PLoS One. 2015 Jun 5;10(6):e0127173. doi: 10.1371/journal.pone.0127173 (PMC4457878; doi:10.1371/journal.pone.0127173)
Supplement: S6 Table — (DOCX) [file pone.0127173.s008.docx]

**Table S6.** Life Expectancy: Regression Results Controlling for GINI and Individualism

| Life Expectancy | Model 1 | | | Model 2 | | | Model 3 | | | Model 4 | | |
| --- | --- | --- | --- | --- | --- | --- | --- | --- | --- | --- | --- | --- |
|  | *B* | *SE B* | *β* | *B* | *SE B* | *β* | *B* | *SE B* | *β* | *B* | *SE B* | *β* |
| GINI | -.04 | .11 | -.07 | .09 | .12 | .16 | .10 | .12 | .17 | .02 | .10 | .03 |
| Individualism |  |  |  | .08 | .04 | .44* | .09 | .04 | .47* | .05 | .04 | .26 |
| Tightness |  |  |  |  |  |  | .11 | .33 | .07 | 4.49 | 1.10 | 2.77** |
| Tightness^2^ |  |  |  |  |  |  |  |  |  | -.33 | .08 | -2.84** |
| df1, df2 | 1, 29 | | | 2, 28 | | | 3, 27 | | | 4, 26 | | |
| *F* | .13 | | | 2.36 | | | 1.56 | | | 6.07** | | |
| *R^2^* | .01 | | | .14 | | | .15 | | | .48 | | |
| *R^2^* Change |  | | | .13 | | | .01 | | | .33 | | |
| *F* for *R^2^* Change |  | | | 4.57* | | | .12 | | | 16.84** | | |

** p* < .05. ** *p* < .01. § *p* < .10.
