# Supplementary material for: Culture and National Well-Being: Should Societies Emphasize Freedom or Constraint?
Source: PLoS One. 2015 Jun 5;10(6):e0127173. doi: 10.1371/journal.pone.0127173 (PMC4457878; doi:10.1371/journal.pone.0127173)
Supplement: S7 Table — (DOCX) [file pone.0127173.s009.docx]

**Table S7.** Male Mortality Rate for Cardiovascular Diseases and Diabetes: Regression Results Controlling for GINI and Individualism

| Mortality Rate: Cardio Diseases and Diabetes, Men | Model 1 | | | Model 2 | | | Model 3 | | | Model 4 | | |
| --- | --- | --- | --- | --- | --- | --- | --- | --- | --- | --- | --- | --- |
|  | *B* | *SE B* | *β* | *B* | *SE B* | *β* | *B* | *SE B* | *β* | *B* | *SE B* | *β* |
| GINI | -.06 | 3.36 | -.01 | -4.13 | 3.70 | -.23 | -4.81 | 3.56 | -.27 | -2.50 | 2.88 | -.14 |
| Individualism |  |  |  | -2.55 | 1.20 | -.44* | -3.64 | 1.29 | -.63** | -2.44 | 1.06 | -.42* |
| Tightness |  |  |  |  |  |  | -18.24 | 9.77 | -.36§ | -147.19 | 32.71 | -2.92** |
| Tightness^2^ |  |  |  |  |  |  |  |  |  | 9.58 | 2.36 | 2.69** |
| df1, df2 | 1, 28 | | | 2, 27 | | | 3, 26 | | | 4, 25 | | |
| *F* | .01 | | | 2.25 | | | 2.80§ | | | 7.47** | | |
| *R^2^* | .01 | | | .14 | | | .24 | | | .54 | | |
| *R^2^* Change |  | | | .13 | | | .10 | | | .30 | | |
| *F* for *R^2^* Change |  | | | 4.51* | | | 3.48§ | | | 16.46** | | |

** p* < .05. ** *p* < .01. § *p* < .10.
