# Supplementary material for: Culture and National Well-Being: Should Societies Emphasize Freedom or Constraint?
Source: PLoS One. 2015 Jun 5;10(6):e0127173. doi: 10.1371/journal.pone.0127173 (PMC4457878; doi:10.1371/journal.pone.0127173)
Supplement: S8 Table — (DOCX) [file pone.0127173.s010.docx]

**Table S8.** Female Mortality Rate for Cardiovascular Diseases and Diabetes: Regression Results Controlling for GINI and Individualism

| Mortality Rate: Cardio Diseases and Diabetes, Women | Model 1 | | | Model 2 | | | Model 3 | | | Model 4 | | |
| --- | --- | --- | --- | --- | --- | --- | --- | --- | --- | --- | --- | --- |
|  | *B* | *SE B* | *β* | *B* | *SE B* | *β* | *B* | *SE B* | *β* | *B* | *SE B* | *β* |
| GINI | 1.92 | 2.24 | .16 | -1.56 | 2.33 | -.13 | -1.82 | 2.33 | -.15 | -.30 | 1.88 | -.03 |
| Individualism |  |  |  | -2.18 | .76 | -.56** | -2.60 | .85 | -.67** | -1.81 | .70 | -.47 |
| Tightness |  |  |  |  |  |  | -6.98 | 6.40 | -.21 | -91.56 | 21.42 | -2.69** |
| Tightness^2^ |  |  |  |  |  |  |  |  |  | 6.29 | 1.55 | 2.62** |
| df1, df2 | 1, 28 | | | 2, 27 | | | 3, 26 | | | 4, 25 | | |
| *F* | .74 | | | 4.63* | | | 3.50* | | | 8.33** | | |
| *R^2^* | .03 | | | .26 | | | .29 | | | .57 | | |
| *R^2^* Change |  | | | .23 | | | .03 | | | .28 | | |
| *F* for *R^2^* Change |  | | | 8.33** | | | 1.19 | | | 16.52** | | |

* *p* < .05. ** *p* < .01. § *p* < .10.
