# Supplementary material for: Culture and National Well-Being: Should Societies Emphasize Freedom or Constraint?
Source: PLoS One. 2015 Jun 5;10(6):e0127173. doi: 10.1371/journal.pone.0127173 (PMC4457878; doi:10.1371/journal.pone.0127173)
Supplement: S9 Table — (DOCX) [file pone.0127173.s011.docx]

**Table S9.** GDP per Capita: Regression Results Controlling for GINI and Individualism

| GDP per capita | Model 1 | | | Model 2 | | | Model 3 | | | Model 4 | | |
| --- | --- | --- | --- | --- | --- | --- | --- | --- | --- | --- | --- | --- |
|  | *B* | *SE B* | *β* | *B* | *SE B* | *β* | *B* | *SE B* | *β* | *B* | *SE B* | *β* |
| GINI | -175.72 | 356.61 | -.09 | 313.56 | 384.61 | .16 | 363.63 | 382.43 | .19 | 205.93 | 365.68 | .11 |
| Individualism |  |  |  | 307.19 | 124.94 | .49* | 386.72 | 138.45 | .62** | 305.17 | 135.06 | .49* |
| Tightness |  |  |  |  |  |  | 1337.44 | 1049.75 | .24 | 10129.86 | 4158.01 | 1.85* |
| Tightness^2^ |  |  |  |  |  |  |  |  |  | -653.46 | 300.25 | -1.69* |
| df1, df2 | 1, 29 | | | 2, 28 | | | 3, 27 | | | 4, 26 | | |
| *F* | .24 | | | 3.17§ | | | 2.70§ | | | 3.49* | | |
| *R^2^* | .01 | | | .18 | | | .23 | | | .35 | | |
| *R^2^* Change |  | | | .17 | | | .05 | | | .12 | | |
| *F* for *R^2^* Change |  | | | 6.05* | | | 1.62 | | | 4.74* | | |

* *p* < .05. ** *p* < .01. § *p* < .10.
