# Supplementary material for: Culture and National Well-Being: Should Societies Emphasize Freedom or Constraint?
Source: PLoS One. 2015 Jun 5;10(6):e0127173. doi: 10.1371/journal.pone.0127173 (PMC4457878; doi:10.1371/journal.pone.0127173)
Supplement: S10 Table — (DOCX) [file pone.0127173.s012.docx]

**Table S10.** Political Instability Index: Regression Results Controlling for GINI and Individualism

| Political Instability  Index | Model 1 | | | Model 2 | | | Model 3 | | | Model 4 | | |
| --- | --- | --- | --- | --- | --- | --- | --- | --- | --- | --- | --- | --- |
|  | *B* | *SE B* | *β* | *B* | *SE B* | *β* | *B* | *SE B* | *β* | *B* | *SE B* | *β* |
| GINI | .03 | .03 | .15 | -.02 | .03 | -.11 | -.03 | .03 | -.15 | -.01 | .03 | -.06 |
| Individualism |  |  |  | -.03 | .01 | -.51* | -.04 | .01 | -.73** | -.03 | .01 | -.58** |
| Tightness |  |  |  |  |  |  | -.22 | .09 | -.44* | -1.16 | .32 | -2.35** |
| Tightness^2^ |  |  |  |  |  |  |  |  |  | .07 | .02 | 2.02** |
| df1, df2 | 1, 29 | | | 2, 28 | | | 3, 27 | | | 4, 26 | | |
| *F* | .70 | | | 3.73* | | | 5.02** | | | 7.23** | | |
| *R^2^* | .02 | | | .21 | | | .36 | | | .53 | | |
| *R^2^* Change |  | | | .19 | | | .15 | | | .17 | | |
| *F* for *R^2^* Change |  | | | 6.62* | | | 6.21* | | | 9.27** | | |

* *p* < .05. ** *p* < .01. § *p* < .10.
