# Supplementary material for: Culture and National Well-Being: Should Societies Emphasize Freedom or Constraint?
Source: PLoS One. 2015 Jun 5;10(6):e0127173. doi: 10.1371/journal.pone.0127173 (PMC4457878; doi:10.1371/journal.pone.0127173)
Supplement: S11 Table — (DOCX) [file pone.0127173.s013.docx]

**Table S11.** Well-Being Composite Score: Regression Results Controlling for GINI and Individualism

| Well-Being  Index | Model 1 | | | Model 2 | | | Model 3 | | | Model 4 | | |
| --- | --- | --- | --- | --- | --- | --- | --- | --- | --- | --- | --- | --- |
|  | *B* | *SE B* | *β* | *B* | *SE B* | *β* | *B* | *SE B* | *β* | *B* | *SE B* | *β* |
| GINI | -.02 | .37 | -.01 | .45 | .41 | .23 | .52 | .40 | .26 | .25 | .31 | .12 |
| Individualism |  |  |  | .30 | .13 | .45* | .40 | .14 | .62* | .26 | .12 | .40* |
| Tightness |  |  |  |  |  |  | 1.79 | 1.09 | .31 | 16.98 | 3.55 | 2.99** |
| Tightness^2^ |  |  |  |  |  |  |  |  |  | -1.13 | .26 | -2.82** |
| df1, df2 | 1, 29 | | | 2, 28 | | | 3, 27 | | | 4, 26 | | |
| *F* | .002 | | | 2.49§ | | | 2.65** | | | 8.19** | | |
| *R^2^* | <.0001 | | | .15 | | | .23 | | | .56 | | |
| *R^2^* Change |  | | | .15 | | | .08 | | | .33 | | |
| *F* for *R^2^* Change |  | | | 4.98* | | | 2.68 | | | 19.37** | | |

* *p* < .05. ** *p* < .01. § *p* < .10.
