# Supplementary material for: Culture and National Well-Being: Should Societies Emphasize Freedom or Constraint?
Source: PLoS One. 2015 Jun 5;10(6):e0127173. doi: 10.1371/journal.pone.0127173 (PMC4457878; doi:10.1371/journal.pone.0127173)
Supplement: S12 Table — (DOCX) [file pone.0127173.s014.docx]

**Table S12.** Corrected Akaike Information Criteria Comparisons

|  | AICc for Linear Model | AICc for Quadratic Model |
| --- | --- | --- |
| Happiness | 183.02 | 179.31 |
| Dysthymia | -158.79 | -161.63 |
| Life Expectancy | 102.90 | 87.44 |
| Suicide | 121.29 | 119.13 |
| Cardiovascular Diseases  and Diabetes Mortality Rate, Men | 313.08 | 297.87 |
| Cardiovascular Diseases  and Diabetes Mortality Rate, Women | 288.90 | 274.93 |
| GDP per Capita | 623.30 | 618.49 |

| Political Instability Index | 26.53 | 18.03 |
| --- | --- | --- |

| Composite Score | 180.89 | 163.34 |
| --- | --- | --- |

Lower AICc values indicate better fit.
